# Early detection and brief intervention for hazardous and harmful drinkers in primary health care in Italy: evaluation of the strategies, activities and experiences of the Istituto Superiore di Sanità (ISS)

**DOI:** 10.1186/1940-0640-7-S1-A30

**Published:** 2012-10-09

**Authors:** Emanuele Scafato, Silvia Ghirini, Aula Rossi, Lucia Galluzzo, Sonia Martire, Lucilla Di Pasquale, Claudia Gandin

**Affiliations:** 1 National Center for Epidemiology, Surveillance and Health Promotion, Rome, Italy

## 

Provisions for early identification and brief intervention (EIBI) for hazardous and harmful drinking in primary care practice were explicitly included in the Italian 2000-2003 National Health Plan. Since that time, a number of strategies were carried out under the framework of different national and international programs. The need for specific training standards on EIBI outlined by the Primary Health Care European Project on Alcohol (PHEPA) were the impetus for the Italian national program “Gaining Health” with the purpose of developing, implementing, and disseminating a national EIBI training program adhering to PHEPA standards. National EIBI courses started in 2007 with the first formal “training the trainers” program opened to general practitioners and other professionals involved in primary care. Courses were funded by the Italian Ministry of Health and the Presidency of the Council of Ministers’ Department of Anti-Drug Policies. Since 2007, six formal courses have been conducted at the ISS and at other regional training sites. The last course was offered in May 2011, and data analysis of training effectiveness is now in progress. The main characteristics of participants from the first five ISS EIBI training courses were as follows: average age, 47.9 years (range, 24-60); gender distribution, men = 38% and women = 62%; professional categories, physicians = 63% and psychologists = 37%. Final analysis of a standardized evaluation form completed by 145 professionals who completed the EIBI program will provide in-depth insight into the effectiveness of the training.

